# Is enhancing cGMP-PKG signalling a promising therapeutic target for heart failure with preserved ejection fraction?

**DOI:** 10.1007/s12471-016-0814-x

**Published:** 2016-02-29

**Authors:** Á. Kovács, A. Alogna, H. Post, N. Hamdani

**Affiliations:** Department of Cardiovascular Physiology, Ruhr University Bochum, Bochum, Germany; Department of Cardiology, Charité Berlin Campus Virchow, Berlin, Germany

**Keywords:** Heart failure with preserved ejection fraction, Diastolic stiffness, cGMP-PKG, Titin, Oxidative stress, Comorbidities

## Abstract

Heart failure with preserved ejection fraction, i.e. HFpEF, is highly prevalent in ageing populations, accounting for more than 50 % of all cases of heart failure in Western societies, and is closely associated with comorbidities such as obesity, diabetes and arterial hypertension. However, all large multicentre trials of potential HFpEF treatments conducted to date have failed to produce positive outcomes. These disappointing results suggest that a ‘one size fits all’ strategy may be ill-suited to HFpEF and support the use of tailored, personalised therapeutic approaches with specific treatments designed for specific comorbidity-related HFpEF phenotypes. The accumulation of a multitude of cardiovascular comorbidities over time leads to increased systemic inflammation, oxidative stress and coronary microvascular endothelial inflammation, eventually resulting in degradation of cyclic guanosine monophosphate (cGMP) via multiple pathways, thereby reducing protein kinase G (PKG) activity. The importance of cGMP-PKG pathway modulation is supported by growing evidence that suggests that this pathway may be a promising therapeutic target, evidence that is mainly based on its role in the phosphorylation of the giant cytoskeletal protein titin. This review will focus on the preclinical and early clinical evidence in the field of cGMP-enhancing therapies and PKG activation.

## Introduction

**Fig. 1:**
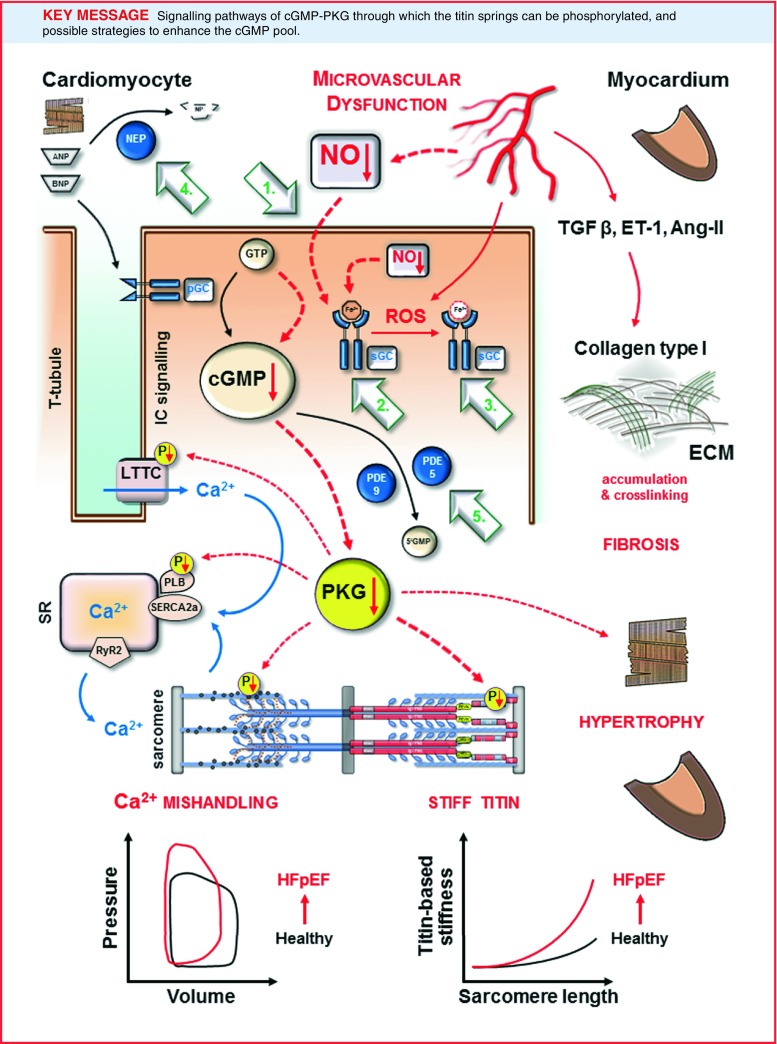
Classes of drugs that modulate the cGMP-PKG pathway within the cardiomyocyte. Cyclic guanosine monophosphate (*cGMP*) is produced either via cytosolic soluble guanylate cyclase (*sGC*), which is activated by nitric oxide (*NO*) or by the transmembrane particulate guanylate cyclase (*pGC*), which is activated by the natriuretic peptides (ANP, BNP). Class of NO and nitroxyl donors is displayed by *No. 1*. sGC stimulators (*No. 2*) target only non-oxidised sGC (Fe^2+^); *vice versa* sGC activators (*No. 3*) target oxidised sGC (Fe^3+^) by reactive oxygen species (*ROS*). Inhibitors of neprilysin (NEP) responsible for ANP and BNP breakdown are indicated by *No. 4*. Phosphodiesterase 5 (PDE5) operates the breakdown of cGMP produced by sGC, while PDE9 is responsible for the breakdown of cGMP produced by pGC (*No. 5*). *5’GMP* guanosine 5’-monophosphate, *Ang-II* angiotensin II, *Ca* ^*2*+^ calcium, *ECM* extracellular matrix, *ET-1* endothelin-1, *GTP* guanosine triphosphate, *HFpEF* heart failure with preserved ejection fraction, *IC* intracellular, *LTCC* L-type calcium channel, *P* phosphate group, *PLB* phospholamban, *PKG* protein kinase G, *SERCA2a* sarco/endoplasmic reticulum Ca^2+^-ATPase, *SR* sarco/endoplasmic reticulum, *RyR2* ryanodine receptor 2, *TGF β* transforming growth factor β.

Greater molecular understanding of cardiac mechanotransduction in normal and failing hearts has provided novel perspectives on the role of cyclic guanosine monophosphate (cGMP)-protein kinase G (PKG) signalling in heart failure with preserved ejection fraction, i.e. HFpEF ([1–5]; Fig. 1).

While initial descriptions of HFpEF focused on left ventricular (LV) diastolic dysfunction, it is now clear that HFpEF actually involves a complex interplay of factors, including LV and vascular stiffness, left atrial impairment, chronotropic incompetence and decreased pulmonary vascular capacitance [6]. Recent echocardiographic speckle tracking studies have shown that global LV function in HFpEF suffers from a loss of longitudinal shortening that is compensated by radial and circumferential shortening [6], together with a loss of LV twist and untwist during systole and diastole, thus indicating damage primarily to the subendocardial muscle fibres. HFpEF is now understood to have a characteristic set of features including LV hypertrophy, concentric remodelling, increased extracellular matrix (ECM), abnormal calcium handling, abnormal relaxation and filling and decreased diastolic distensibility ([6]; Fig. 1). Diastolic function is often conceptualised as the totality of an active process of pressure decay (relaxation) during early diastole, which is related to myofilament dissociation and calcium reuptake, and to a ‘passive’ stiffness dependent on viscoelastic properties, and modulated by mechanical changes via the sarcomere, ECM, chamber or pericardium [6]. By contrast, diastolic abnormalities in HFpEF include delayed early relaxation, myocardial and myocyte stiffening, and associated changes in filling dynamics ([6]; Fig. 1).

In diastole, the ECM contributes to passive stiffness, helping to prevent overstretch, myocyte slippage and tissue deformation during ventricular filling. ECM components also serve as modulators of growth and tissue differentiation. Certain forms of excessive collagen deposition are associated with the chronic pressure overload that occurs in hypertensive heart disease and aortic stenosis, leading to an increase in myocardial stiffness. Collagen is a major determinant of ECM-based stiffness, and factors such as total collagen levels, the expression of collagen type I relative to type 3 [7], and the degree of collagen crosslinking are all increased in HFpEF and aortic stenosis (Fig. 1), and have been linked to diastolic LV dysfunction [8–10]. In addition, myocardial collagen deposition in HFpEF results from the differentiation of fibroblasts into myofibroblasts due to release of transforming growth factor β by monocytes migrating through the inflamed microvascular endothelium ([11, 12]; Fig. 1). Microvascular inflammation also favours the proliferation of fibroblasts and myofibroblasts as a direct consequence of reduced nitric oxide (NO) bioavailability and the subsequent unopposed profibrotic actions of growth-promoting hormones such as endothelin-1, angiotensin II and aldosterone ([11, 12]; Fig. 1).

## Oxidative stress-mediated cell signalling in comorbidities associated with HFpEF

Patients with HFpEF show characteristic features, being generally elderly, more often female, and display a high prevalence of non-cardiac comorbidities such as overweight/obesity, hypertension, diabetes, chronic obstructive pulmonary disease, anaemia and chronic kidney disease. Interestingly, systemic inflammation and endothelial dysfunction are important hallmarks of these comorbidities, and may also drive myocardial dysfunction and remodelling in HFpEF through coronary microvascular endothelial inflammation [11, 12]. This latter effect is evident in the reduced aortic distensibility, higher arterial load and deficient vasodilatory reserve seen in HFpEF, which may be due to upregulation of endothelial NO synthase (NOS) [11, 12]. It has been suggested that control of cardiovascular risk factors and comorbidities in HFpEF, such as body mass index, smoking and atrial fibrillation, may provide a successful therapeutic strategy as these have been identified as traditional triggers for new-onset HFpEF; these factors are therefore likely to be important in the prevention and treatment of HFpEF [12, 13].

Considerable evidence now supports a role for oxidative stress and inflammation in HFpEF progression, in addition to the endothelial dysfunction that is common to many forms of vascular disease [12, 14, 15]. Endothelial dysfunction is thought to be independent of a specific aetiology and vascular structure, and may be due to altered oxidative stress processes that result in impaired NO function [12, 14, 15]. Oxidative stress-mediated downregulation of NO-cGMP-PKG signalling has been demonstrated in various experimental models of diabetes, insulin resistance, obesity and metabolic syndrome [1–4, 12, 14, 15]. Reduced NO function has been attributed to deficiencies in the levels or function of NOS signalling enzymes, as well as scavenging effects due to an enhanced production of superoxide anion or angiotensin II [16, 17]. All of these changes may impair cGMP-PKG signalling, potentially explaining its diminished function in HFpEF and suggesting a potential therapeutic target for this specific form of heart disease (Fig. 1).

## cGMP signalling in the cardiomyocyte

cGMP is synthesised by two distinct pathways: the first requires activation of soluble guanylate cyclase (sGC) by NO, while the second requires particulate guanylate cyclase (pGC) coupled to natriuretic peptide (NP) ([18, 19]; Fig. 1). Well-characterised downstream signalling effectors of cGMP include cGMP-dependent PKG, cGMP-susceptible phosphodiesterases (PDEs), and cGMP-gated cation channels (Fig. 1). PKG phosphorylates a vast number of target proteins, exerting a wide range of downstream effects such as inhibition of the L-type calcium channel, enhancement of intracellular diastolic calcium reuptake through phosphorylation of phospholamban, suppression of hypertrophic signalling through inhibition of G-protein coupled receptors and the transient receptor potential canonical channel, inhibition of ischaemia-reperfusion injury through phosphorylation of the ATP-sensitive potassium channel and stimulation of LV relaxation and distensibility by phosphorylation of troponin I (TnI) and titin ([19, 20]; Fig. 1).

## cGMP and HFpEF pathophysiology

One finding of potential clinical importance was the favourable reaction of slow LV relaxation and high diastolic LV stiffness in HFpEF to increased PKG activity following *in vivo* administration of the PDE5A inhibitor sildenafil, which facilitates increased myocardial PKG activity by inhibiting the breakdown of cGMP by PDE5A [1–5]. This is significant, because relative to both aortic stenosis and heart failure with reduced ejection fraction (HFrEF) [5], HFpEF patients show reduced myocardial PKG activity and lower cGMP concentrations, both of which are associated with increased titin-based stiffness, titin hypophosphorylation and elevated myocardial nitrotyrosine levels, indicative of raised nitrosative/oxidative stress [5]. Another important finding was the demonstration of the reduced bioavailability of NO in HFpEF patients and in an HFpEF animal model, suggesting that impaired cGMP-PKG signalling in HFpEF is related to the low myocardial NO bioavailability that results from high nitrosative/oxidative stress [21]. All of these findings were associated with titin hypophosphorylation (Fig. 1). A further related finding was that the accumulation of a variety of cardiovascular comorbidities such as ageing, hypertension, diabetes, obesity and physical inactivity (which limit NO bioavailability and thereby decrease PKG activity in adjacent cardiomyocytes) has been mechanistically linked to the classical hallmarks of LV stiffness and diastolic dysfunction, which include concentric LV remodelling and cardiomyocyte stiffening due to titin hypophosphorylation [1–5, 10, 12, 19, 22]. Taken together, these findings suggest that PKG-mediated phosphorylation of titin could be a therapeutic target in HFpEF, although the elements of the NO-cGMP-PKG signalling network most critical to titin phosphorylation and stiffness still need to be elucidated.

## Is PDE inhibition beneficial for HFpEF?

The relevance of the NO-cGMP-PKG signalling network is further emphasised by the actions of sildenafil in many studies. Inhibition of cGMP degradation enhances NO-mediated vasodilation by sildenafil, restores LV relaxation kinetics in mice exposed to transverse aortic constriction [23], and reduces diastolic LV stiffness in an old hypertensive dog model through restored phosphorylation of titin [4]. Similar effects have been observed in patients with HFrEF and in HFpEF patients with pulmonary hypertension [24–26]. PDE5A inhibitors have a range of beneficial effects and can attenuate adrenergic stimulation, reduce ventricular-vascular stiffening, improve endothelial function, reduce pulmonary vascular resistance and enhance exercise tolerance in HFrEF [24–26].

Given the known properties of sildenafil, a long-term (24-week) trial of sildenafil (RELAX trial) in patients with HFpEF reported an unexpected outcome [27], producing no evidence for an elevation of the low cGMP content expected in HFpEF following inhibition of PDE5A. Indeed, in the > 100 HFpEF patients studied, sildenafil failed to raise plasma cGMP or ameliorate diastolic LV dysfunction. The authors suggested that these disappointing results were (partially) attributable to the relatively low right-sided heart pressures in their patient group compared with earlier studies in HFrEF. In addition, elevated basal plasma levels of N-terminal pro-BNP (NT-proBNP) and the high prevalence of atrial fibrillation in these patients suggested that they were already at an advanced stage of HFpEF and therefore less likely to benefit from a strategy limited to the inhibition of cGMP breakdown via PDE5A. On the other hand, this negative outcome may also be due, in part, to the fact that PDE5A is expressed at low levels in heart failure and myocardial tissues. One explanation for the results described above was hinted at recently when it was reported that cGMP-specific PDE9A regulates cGMP signalling independently of NO and contributes to stress-induced cardiac disease. Interestingly, PDE9A is increased in hypertrophied hearts and in HFpEF patients [28], possibly suggesting that continuing expression of PDE9A may have contributed to the neutral outcome of the RELAX trial. This further suggests that, in addition to PDE5A, PDE9A may also be a *bona fide* therapeutic target in heart failure. Conversely, the failure of the RELAX trial might also be related to the subcellular microdomains of cGMP recruited by inhibition of PDE5A, which are NO dependent and therefore ineffective in recruiting a preload reserve under conditions of reduced NO bioavailability.

In light of the many caveats surrounding the RELAX trial it is worth recalling that other small studies have demonstrated benefits of sildenafil in patients with high blood pressure, right ventricular dysfunction and pulmonary arterial hypertension. In some studies sildenafil was reported to be beneficial for right ventricular function and pulmonary pressures in HFpEF patients with pulmonary hypertension [29]. This is in contrast with a more typical HFpEF population regarding gender and comorbidities, in which sildenafil did not improve invasive right ventricular haemodynamic parameters, nor did it reduce pulmonary arterial pressures when HFpEF was complicated with pulmonary hypertension [30]. Thus, despite these contradictory observations, the possibility remains that sildenafil is effective, but perhaps only in HFpEF sub-populations with a specific patient profile associated with relevant comorbidities. However, many pharmacological strategies for cGMP pathway modulation/enhancement remain, and the effects of an intervention might vary with the mode and pathway site of action. The relative importance of possible interventions remains to be determined.

## PKG activators and strategies to enhance cGMP

One of the abovementioned studies showed that 30 % of HFpEF patients have diastolic dysfunction with no increase in LV collagen [31], but with increased cardiomyocyte stiffness (attributed to titin hypophosphorylation due to cGMP-PKG deterioration) [5]. This finding suggests that, depending on comorbidities, cGMP-PKG and titin may be promising therapeutic targets for heart failure with diastolic abnormalities. Boosting myocardial cGMP levels might be possible by alternative means, which can be achieved through stimulation of pGC activity, through augmentation of sGC activity, enhancement of NOS coupling and NO production or via inhibition of cGMP degrading enzymes. However, this may require the study of possible altered proteins in HFpEF that are involved in the stimulation or degradation of cGMP and which can be affected by oxidative stress (e.g. the expression levels of GC-A and -C receptors, sGC, PDE5A and 9A, and NPs including ANP, BNP and CNP). A better understanding of these factors would help determine which upstream mechanism is responsible for reduced cGMP-PKG, and would help dissect the effect of risk factors on upstream pathways of cGMP-PKG signalling. We expect expression levels of these proteins to be distinctive, dependent on the risk factors. The body of evidence supporting cGMP enhancement and oxidative stress-based comorbidities provides a convincing rationale for further investigation of this pathway in HFpEF, but will probably require the inhibition and/or activation of several cGMP-PKG signalling pathways, and will require the identification of those elements of the NO-cGMP-PKG signalling network that are critical for improvement of diastolic stiffness *in vivo*.

### NO and nitroxyl donors

Previous clinical trials of PDE5A inhibitors, NO donors, sGC activators and neprilysin inhibitors have focused on acute vasodilatory effects. This focus is now shifting towards their potential direct myocardial effects, independent of afterload reduction. Nitroxyl donors, such as 1-nitrosocyclohexyl acetate, may have therapeutic benefits in heart failure due to the direct action of nitroxyl on myofilaments, sarco/endoplasmic reticulum Ca^2+^-ATPase (SERCA2a) and ryanodine receptor 2 (RyR2) [32], which all have overall positive inotropic and lusitropic effects on the heart. Whether nitroxyl donors are beneficial in treating HFpEF still needs to be elucidated. On the other hand, the NO donors increase diastolic cell length [33], inducing earlier LV relaxation *in vitro*. In line with this, in patients with chest pain but no evidence of coronary lesions, intracoronary infusion of sodium nitroprusside resulted in a slightly decreased LV peak systolic pressure and an acute increase of LV end-diastolic capacitance [34, 35]. In theory, the described effects on LV compliance suggest that NO donors would be ideal in tackling LV stiffness in HFpEF patients. However, major limitations of the available NO donors have hindered progress in the field. Firstly, patients with heart failure develop a rapid tolerance phenomenon, also termed tachyphylaxis, leading to an impaired NO bioactivation that is related to increased vascular oxidative stress [6, 12, 13]. Secondly, chronic administration of the NO donor isosorbide mononitrate was shown to increase oxidative stress and induce endothelial dysfunction by increasing the expression of endothelin [36]. Finally, HFpEF patients are sensitive to preload reduction and are therefore more susceptible to haemodynamic instability and stroke volume reduction following vasodilation than HFrEF patients [6, 12, 13]. NO donors remain important therapeutic options for vascular unloading and to decrease filling pressures in acutely decompensated patients, but are less applicable in chronic heart failure. In addition, NO signalling has anti-fibrotic, anti-hypertrophic and anti-adrenergic actions that oppose adverse cardiac remodelling, and thus may represent potential therapeutic strategies for HFpEF [37–39]. Furthermore, tetrahydrobiopterin (BH4) and endothelial NOS activators could be useful in reducing diastolic dysfunction, since the deoxycorticosterone acetate (DOCA)-salt mouse model is associated with BH4 depletion and uncoupled NOS activity, but BH4 administration consistently reduced DOCA-salt-associated myosin binding protein C glutathionylation and diastolic dysfunction [37–39].

### sGC activation and stimulation

The responsiveness of sGC to NO and its subsequent ability to generate cGMP is impaired by oxidation (Fig. 1). NOS can also become uncoupled due to oxidation, resulting in the synthesis of superoxide. sGC activators and stimulators are two recently discovered classes of compounds that modulate sGC activity.

sGC activators are ideal substitutes for NO under conditions of endothelial dysfunction, such as increased oxidative stress [40, 41]. So far, this class of drugs has only been tested in HFrEF patients. In a canine model of congestive heart failure, the sGC activator cinaciguat was shown to potently unload the heart, increasing cardiac output and renal flow without any further neurohormonal activation [40, 41]. Early clinical data in acute decompensated heart failure showed reduction of filling pressure and an increased cardiac output [40, 41]. However, a phase IIb clinical trial (COMPOSE) in a similar population was terminated early because of excessive hypotension in the cinaciguat arm [42].

Stimulators of sGC reduce renal and cardiac organ damage in experimental high and low renin models [43]. Recently, a phase IIb clinical study in patients with pulmonary hypertension caused by systolic LV dysfunction found that the sGC stimulator, riociguat, failed to reduce pulmonary artery pressure, but did cause increases in cardiac index and stroke volume, and improved systemic and pulmonary resistance [44]. Another oral sGC stimulator, vericiguat, is now under investigation in a phase II clinical trial, recruiting both HFpEF and HFrEF patients (SOCRATES-PRESERVED and -REDUCED, respectively) [45]; however, the latest report on the HFrEF study population found no significant effect of vericiguat on NT-proBNP levels at 12 weeks of dose-finding treatments [46].

### NP-mediated cGMP-PKG signalling

ANP and BNP are released by atrial and ventricular cardiomyocytes, respectively (Fig. 1), in response to increased myocardial wall stress due to volume- or pressure-overload conditions, and both induce vasodilation and natriuresis, whereas CNP does not induce natriuresis at physiological concentrations [47]. The vasopeptidase enzyme neprilysin is responsible for the breakdown of natriuretic peptides (Fig. 1). The neprilysin inhibitor, omapatrilat, which is actually a combined inhibitor of angiotensin-converting enzyme and neprilysin, showed promising results in HFrEF, but further investigation of this compound was hindered by excessive angioedema in a subsequent phase III clinical trial [48, 49]. A new compound, LCZ696, which is also a combined angiotensin receptor and neprilysin inhibitor, was recently developed and tested in a phase II HFpEF trial [48, 49]. The compound achieved a greater reduction of NT-proBNP at 12 weeks compared with valsartan. In addition, follow-up at 36 weeks showed a greater reduction of left atrial volume index, and improvements of New York Heart Association functional class and estimated glomerular filtration rates [50, 51]. Of note, all these changes were independent of reduction in systolic blood pressure. A phase III clinical trial in HFpEF patients is currently under way and will clarify whether these preliminary findings translate to improved outcomes.

### PDEs inhibitors: possible therapeutic targets for HFpEF

As previously discussed, inhibitors of cGMP degrading enzymes, in particular of PDE5A and PDE9A (some of which are currently approved for treatment of pulmonary arterial hypertension), enhance cGMP-driven effects and consequently increase cardiac muscle relaxation via PKG-mediated phosphorylation of TnI and titin ([1–5, 10, 19, 28, 31]; Fig. 1). A role for PDE5A in LV remodelling has been proposed in a study by Takimoto on PDE5A knockout mice [23]. In addition, administration of the PDE5A inhibitor sildenafil increased LV compliance in a canine model of LV concentric hypertrophy, supporting the possible translation of these findings to patients. Sildenafil was also reported to increase LV capacitance in HFpEF patients with pulmonary hypertension [24–26, 29], in contrast with the outcome of the RELAX trial [27], the negative outcome of which may be related to factors other than those proposed and discussed by the authors. Perhaps the first issue to consider is that PDE5A was shown to be upregulated in the left ventricle of end-stage HFrEF patients, but never in HFpEF patients. PDE5A inhibitors seem to be more effective in reducing right ventricular load in pulmonary hypertension than in HFpEF. A recent study has now shifted attention to the PDE9A isoform [28], which seems to control a pool of cGMP (located at the T-tubular invagination of the plasma membrane) that is produced by the ANP-pGC pathway and independent of NO (Fig. 1).

## Summary and Conclusions

HFpEF is a major public health problem that lacks effective evidence-based therapies. The cGMP pathway plays a central role in the derangements integral to HFpEF pathophysiology. The capacity of PKG to phosphorylate titin and lower titin-based stiffness has formed the basis for several therapeutic interventions that activate this pathway. The body of evidence surrounding cGMP enhancement thus provides a compelling rationale for further investigation of this pathway in HFpEF. Current evidence also suggests that the cGMP-PKG deficiency seen in HFpEF is a result of a wider pro-inflammatory state that is accompanied by widespread endothelial dysfunction and oxidative stress. This then leads to a reduction in or inactivation of several key signalling pathways, which subsequently affects the pool of cGMP. These changes are not only attributable to an increased rate of cGMP degradation via PDE5A, but may also be partly dependent on the interplay of several signalling pathways. Research priorities therefore include the elucidation of the key signalling pathways that lead to elevated cGMP, increased titin phosphorylation and reduced passive stiffness in cardiomyocytes. A better understanding of these key issues will have direct consequences when considering their relative therapeutic potentials for improving diastolic function.

### Funding

DFG (HA 7512/2 − 1), MERCUR (GZ An-2015-0031), FoRUM (F808N-14), FoRUM (Az. F765-13).
